# Nitric Oxide Synthase is Necessary for Normal Urogenital Development

**DOI:** 10.4172/2167-0250.1000108

**Published:** 2013-11-04

**Authors:** Christopher Bond, Omer Onur Cakir, Kevin T. McVary, Carol A. Podlasek

**Affiliations:** 1 Department of Urology, Northwestern University, Feinberg School of Medicine, Chicago, IL, USA; 2 Division of Urology, Southern Illinois University School of Medicine, Springfield, IL, USA

**Keywords:** NOS, Development, Penis, Prostate, Bladder, Urogenital

## Abstract

**Introduction:**

Neuronal nitric oxide synthase (NOS-I) is significantly decreased with Cavernous Nerve (CN) injury in Erectile Dysfunction (ED) models. Increased apoptosis and collagen deposition accompany decreased NOS/CN injury, however these changes are typically attributed to the altered signaling of other factors, and a contribution of NOS in maintenance of urogenital structures has not previously been examined. Morphological changes in the corpora cavernosa occur at the same time as decreased NOS, suggesting a potential connection between decreased/inhibited NOS and morphological changes associated with ED. In this study we propose that NOS impacts urogenital morphology during development and will examine this hypothesis by NOS inhibition with L-NAME.

**Methods:**

Primary outcomes were H&E, western and TUNEL to determine if penis, prostate and bladder morphology were altered with L-NAME treatment of Postnatal day 4 (P4) Sprague Dawley rats for 8 days. Tissue weight and immunohistochemical analysis for NOS were performed. Secondary evaluation of NOS-I regulation by Sonic Hedgehog (SHH) was examined by SHH inhibition in the pelvic ganglia (PG) and NOS-I protein was quantified by western in the PG/CN and penis. Nos abundance was quantified by RT-PCR during urogenital development and after CN injury.

**Results:**

Apoptosis increased and penis, prostate and bladder morphology were altered with L-NAME. NOS inhibition decreased bladder weight 25%. SHH inhibition decreased NOS-I 35% in the PG/CN and 47% in the penis. Nos-III expression spiked within the first two weeks after birth in the penis but remained abundant in the adult. In the prostate, Nos-III was abundant immediately after birth and declined steadily with age. Nos-I expression in the PG/CN decreased sharply with CN injury and returned to baseline by 7 days.

**Conclusions:**

NOS is required for normal urogenital development. Since NOS is decreased with ED, it may contribute to the abnormal morphology observed in ED patients and animal models.

## Introduction

Nitric oxide (NO) is a widely distributed neurotransmitter. Several studies have examined the function of NO as a critical mediator of smooth muscle relaxation in adult urogenital organs, including the penis, prostate, and bladder. Nitric oxide is synthesized from L-arginine by Nitric Oxide Synthase (NOS) in non-adrenergic/non-cholinergic nerve terminals and in endothelium. There are three isoforms of the enzyme, which are, neuronal NOS (nNOS, NOS-I), endothelial NOS (eNOS, NOS-III), and inducible NOS (iNOS, NOS-II), which are localized in nerve terminals, endothelium and play a role in the inflammatory response. While smooth muscle relaxation mediated by NOS, has been well documented in the penis and prostate, little is known about the potential function of NO/NOS in differentiation and morphogenesis of the urogenital organs, which takes place primarily in the postnatal period after birth [1,2]. The potential role of NOS in tissue morphogenesis is important because in adult urogenital organs, NOS is decreased with CN injury in prostatectomy, diabetic and aging models. It has been assumed that the only function of NOS is in smooth muscle relaxation, however if NOS contributes to tissue morphogenesis, then decreased NOS may contribute to morphological changes associated with CN injury. In order to test this hypothesis NOS inhibition must be performed in young animals, prior to erectile function and smooth muscle relaxation, so that NOS function can be differentiated.

In the adult penis, NOS-I and III are localized in neuronal and endothelial tissue [3,4]. NOS-I protein was significantly decreased in CN injury, diabetic and aging models of erectile dysfunction [5–9]. In all of these models increased apoptosis and deposition of collagen result in remodeling of the corpora cavernosa, which is an underlying cause of erectile dysfunction (ED) development. Decreased NOS is commonly associated with decreased ability of the smooth muscle to relax, leading to ED, however morphological changes in the corpora cavernosa occur at the same time as decreased NOS, suggesting a potential connection between altered NOS signaling and morphological changes associated with ED. Such interactions between altered NOS signaling and morphological changes has been suggested in the development of Peyronie’s fibrotic plaque in which it has been shown that NOS-II expression reduces collagen deposition and L-NAME treatment of CN injured mice improves erectile function through a mechanism potentially involving apoptosis [10–12]. The significance of these findings is that altered NOS signaling may not only effect relaxation of smooth muscle, it may also play a regulatory role in establishing penile morphology and in maintenance of architecture in other urogenital organs as well.

NOS isoforms are also expressed in the adult prostate and bladder. In the prostate, NOS plays a role in smooth muscle tone and relaxation, neuronal innervations and regulation of vascular, secretory and hormonal pathways [13,14]. The most abundant isoform in the normal prostate is NOS-III [15]. Reports of NOS localization vary in the prostate depending on species and lobe however NOS appears most abundant in nerve fibers and ductal epithelium [15,16]. NOS-III is significantly decreased in diabetic prostate while NOS-I is increased [15]. This is significant since emphasis has been placed on benign prostatic hyperplasia (BPH) as the underlying risk factor for Lower Urinary Tract Symptoms (LUTS), ignoring the potential for altered smooth muscle tone of the prostate. Changes in NOS may affect proliferation and apoptotic indices and thus contribute to LUTS development. This hypothesis is supported by correlation of increased NOS-III and NOS-I with apoptosis induction and antiproliferative effects of NOS in diabetic BB β-cells of the pancreas [17–19].

In the adult bladder, extensive innervation is required for regulation of micturition and NO plays a significant role in this process, with NO donors reducing the contraction frequency in an experimentally induced bladder over-activity model and NOS inhibition reduces micturition volume and bladder capacity [20–22]. Basal urothelial cells of the guinea pig bladder express NOS-I, NOS-I and III were identified in urothelium and interstitial cells of the and neurogenic bladder [23,24]. Both ED and incontinence are frequent complications of prostatectomy and NOS-I was decreased in the bladder after CN injury [25]. Thus morphological changes that occur in the bladder after injury may also be related to changes in NOS signaling.

In this manuscript we examine a novel hypothesis, that NOS may be a regulator of urogenital morphology by examining the potential role of NOS in regulation of postnatal accessory sex organ differentiation and morphogenesis. Thus changes in NOS signaling in response to injury and/or systemic diseases (i.e. diabetes) may be significant for morphological remodeling of the penis, prostate and bladder in addition to its neurally mediated effects on smooth muscle relaxation.

## Materials and Methods

### Animals

Ninety-one Sprague-Dawley rats embryonic day 19 (E19) through postnatal day 120 (P120) were obtained from Charles River. The weight of the rats was 4g–400g.

### Ethics statement

All animals were cared for according to the National Institute of Health Guidelines for the Care and Use of Laboratory Animals and protocols received institutional approval.

### L-NG-Nitroarginine Methyl Ester (L-NAME) injection

L-NAME (6.5 mg, Santa Cruz Biotechnology, Inc, Santa Cruz, CA) was dissolved in phosphate buffered saline (PBS, 1 mL). L-NAME inhibits cGMP formation in endothelial cells and requires hydrolysis of the methyl ester by cellular esterases to become a fully functional inhibitor (L-NNA). L-NAME exhibits some selectivity for NOS inhibition. It has Ki values of 15 nM, 39 nM and 4–65 μM for NOSI, NOSIII, and NOSII, respectively. P4 Sprague Dawley rats were given an intraperitoneal injection of 50 mg/kg/day L-NAME or PBS (control) each day for 8 days. Rats were sacrificed at P12 and penis, prostate and bladder tissue were weighed and frozen for analysis.

### SHH inhibition in the pelvic ganglia

Affi-Gel beads (100–200 mesh, Bio-Rad, Hercules, CA) were equilibrated with 100 μl of 5E1 SHH inhibitor (400 μg/ml, Jessel, Hybridoma Bank University of Iowa) or mouse IgG (control) overnight at 4°C. Approximately 10–20 beads were injected under the PG bilaterally and rats were sacrificed after 2 days. PG/CN and penis from 5E1 (4 rats) and IgG treated (4 rats) rats were homogenized for western analysis.

### Bilateral CN crush

PG/CN were exposed and microforceps (size 0.02×0.06 mm) were used to crush the CN bilaterally for 30 seconds. This method of CN crush has commonly been used in the literature and the extent and reproducibility of crush injury were previously verified [26–28]. Sham surgery (control) was performed by exposing but not crushing the CN (n=2). Rats were sacrificed 1,2,4 and 7 days after injury (n=8) and PG/CN were homogenized for western analysis for NOS-I.

### Immunohistochemical analysis (IHC)

IHC was performed on penis, prostate and bladder as described previously using the Dako LSAB + System, HRP [29]. Sections were incubated with mouse monoclonal antibodies for NOS-I and NOS-III (250 μg/ml, Transduction Laboratories, Lexington, Kentucky). Sections were stained with 3,3′-Diaminobenzidine (DAB) and mounted using DPX Mounting media (Electron Microscopy Sciences, Hatfield, PA).

### Western analysis

PG/CN and penis tissue were homogenized as outlined previously [30]. 100 μg protein were separated via electrophoresis using a 10% polyacrylamide gel and transferred to a nitrocellulose membrane (Bio-Rad) using a mini Trans-Blot electrophoretic transfer cell (Bio-Rad) for 2 hours. Membranes were blocked with 5% nonfat skim milk in PBS-Tween and were incubated with either, 1/1000 mouse NOS-I (BD Transduction), 1/1000 mouse NOS-III (BD Transduction) 1/50,000 mouse β-ACTIN (Sigma), 1/50,000 mouse α-ACTIN (Sigma), 1/1000 rabbit GAPDH (Cell Signaling) antibodies, overnight at 4°C. Membranes were incubated with 1/2000 or 1/80,000 chicken anti-mouse (Santa Cruz) or 1/2000 chicken anti-rabbit (Santa Cruz) secondary antibody for 1.5 hours. Protein bands were visualized using HRP-conjugated anti-biotin (ECL, Amersham), were exposed to hyperfilm and were quantified using Kodak 1D software (Rochester, NY), to determine the ratio of the density of NOS-I/β-ACTIN. Samples were run in duplicate and the results were averaged.

### RT-PCR

Total RNA was isolated from Sprague Dawley rat penis and prostate (E19-P120, n=59) with TRIzol (Invitrogen, Carlsbad, CA) as previously described and was DNAse treated with RQ1 DNAse (Roche, Indiabapolis, IN) to eliminate any genomic DNA contamination [5]. The presence of any remaining DNA contamination was assessed by RT-PCR with the reverse transcriptase enzyme omitted. RT-PCR was performed on 150 ng total RNA (per tube) using the Gene Amp RNA PCR Core kit (Perkin-Elmer, Branchburg, NJ) as described previously [5]. Primers (NosIIIs: *5′-AGG CTG CTG CCC GAG ATA TCT TCA-3′, NosIIIas: 5′-TTG GGT GGG CAC ACA CCT ATG TGG-3′, Gapdhs: 5′-GTC GGT GTC AAC GGA TTT G-3′ and Gapdhas: 5′-ACA AAC ATG GGG GCA TCA G-3′)* were synthesized at the Northwestern University Biotechnology Facility and products were restriction digested to confirm they represented the sequence of interest. Semi-quantitative RT-PCR was performed by determining the ratio of Nos-III/Gapdh in the linear range, as described previously [5]. Assays were performed in triplicate on individual tissue specimens and the product ratios reported as the mean plus or minus the standard error of the mean.

### TUNEL

TUNEL assay for apoptosis was performed according to manufacturer’s instructions using the ApopTag kit (Intergen, Purchase, NY) on control (n=3) and L-NAME treated (n=3) penis, prostate, and bladder as previously described [31].

### Statistical analysis

A t-test was performed to determine significant differences (p ≤ 0.05) and the results were reported ± the standard error of the mean.

## Results

### Postnatal differentiation

**Figure 1:**
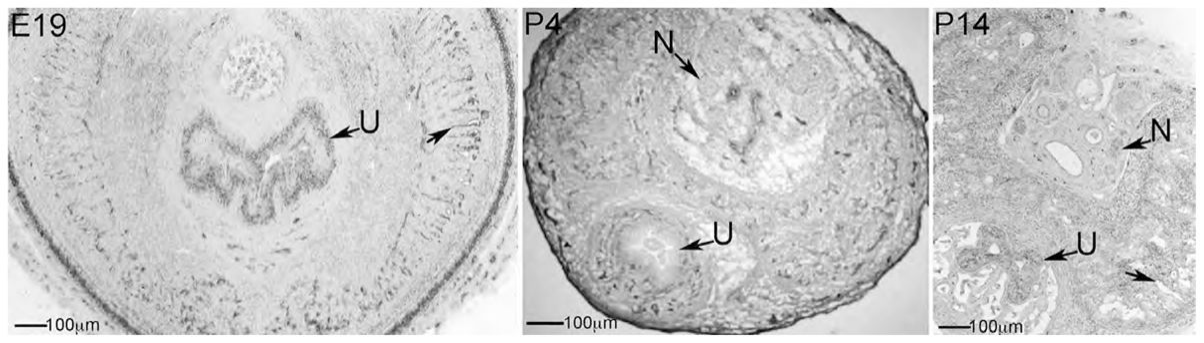
Immunohistochemical analysis of NOS-III protein in E19, P4 and P14 penis. Sinusoidal development of the corpora cavernosa takes place primarily in the postnatal period, during the first weeks after birth. Arrows indicate corpora cavernosal sinuses. N=nerve. U=urethra. 63X magnification.

Substantial development occurs in the postnatal period after birth in urogenital organs as shown by Immunohistochemical (IHC) analysis of NOS-III protein in the rat penis at E19, P4 and P14 (Figure 1). Differentiation into erectile tissue containing both lacunae and trabeculae occurs during the first week after birth as was visualized at P4 (Figure 1) [32]. By P14, cavernous spaces were large, irregularly shaped and lined by an attenuated endothelium (Figure 1) [33]. Cavernae do not resemble the adult configuration until P40 and are exclusively of the adult type by P60 [33].

### NOS regulation by SHH

**Figure 2:**
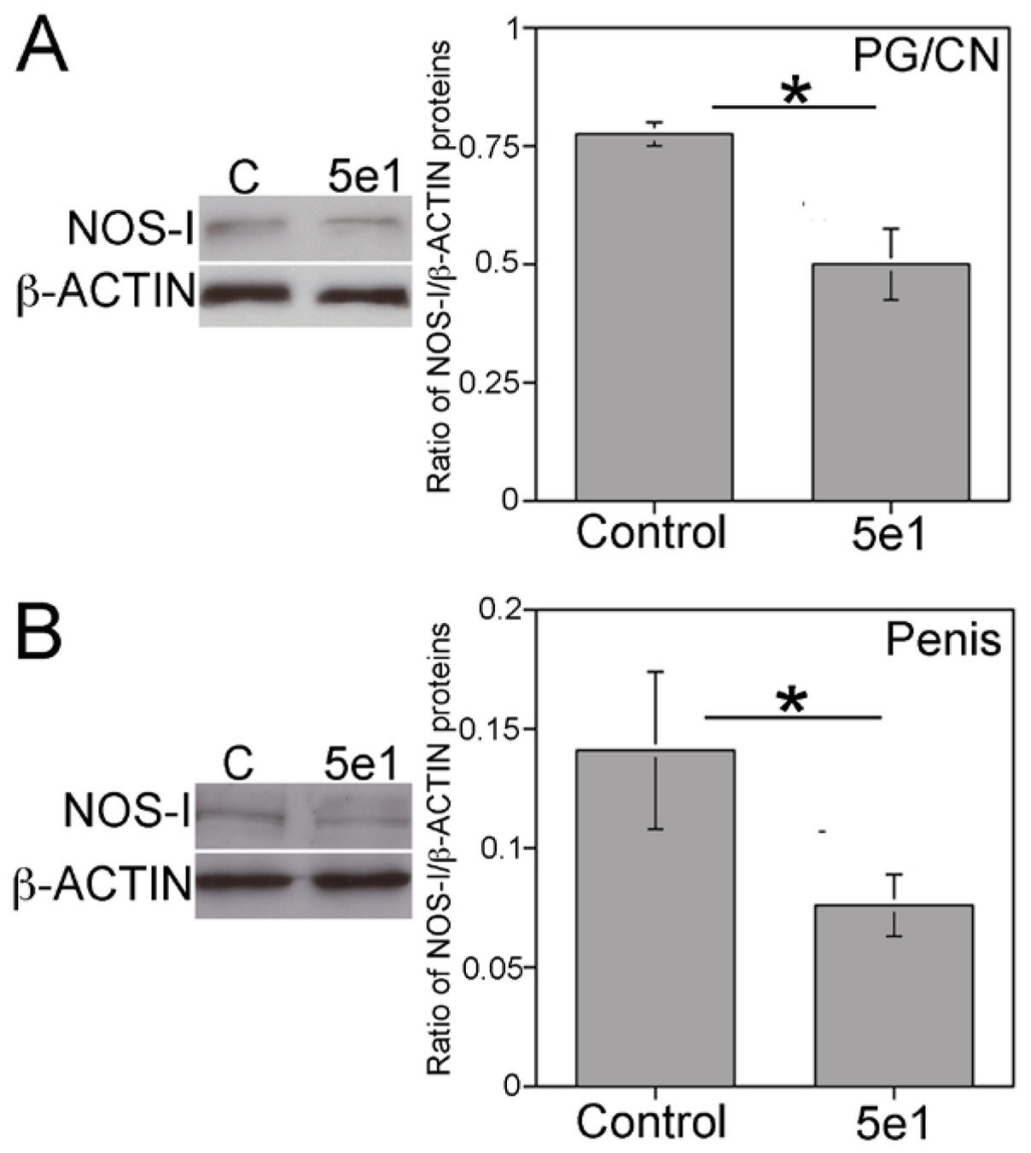
Western analysis of NOS-I in PG/CN and penis of Sprague Dawley rats that were treated with either 5e1 SHH inhibitor or mouse IgG (control) in the pelvic ganglia (PG) via Affi-Gel beads for two days. NOS-I protein decreased 35% in the PG/CN (A, p=0.013) and 47% in the penis (B, p=0.049) in response to SHH inhibition. Asterisks indicate significant differences.

We examined if NOS is a target of SHH signaling by quantifying NOS-I/β-ACTIN by Western analysis in PG/CN and penis tissue of Sprague Dawley rats that were treated with 5e1 SHH inhibitor or mouse IgG (control) via Affi-Gel beads placed under the PG. NOS-I was significantly decreased 35% in the PG/CN with SHH inhibition (p=0.013) and 47% in the penis (p=0.049) in comparison to controls (Figure 2).

### Penis, prostate and bladder weights with L-NAME treatment

**We** examined if NOS plays a role in postnatal development of urogenital tissues by treating P4 rats with L-NAME (NOS inhibitor) for 8 days. The wet weight of penis, prostate and bladder tissues was measured at the end of L-NAME treatment in P12 Sprague Dawley rats. P4-P12 was chosen as the time frame for analysis of NOS inhibition since profound urogenital differentiation/development takes place during the first two weeks after birth [33]. Penis and prostate weights were unchanged with L-NAME treatment (n=4) in comparison to controls (n=4, p=0.19 and 0.32 respectively) (Table 1). However, bladder weight increased 25% in L-NAME treated rats (p=0.018) (Table 1).

**Figure 3:**
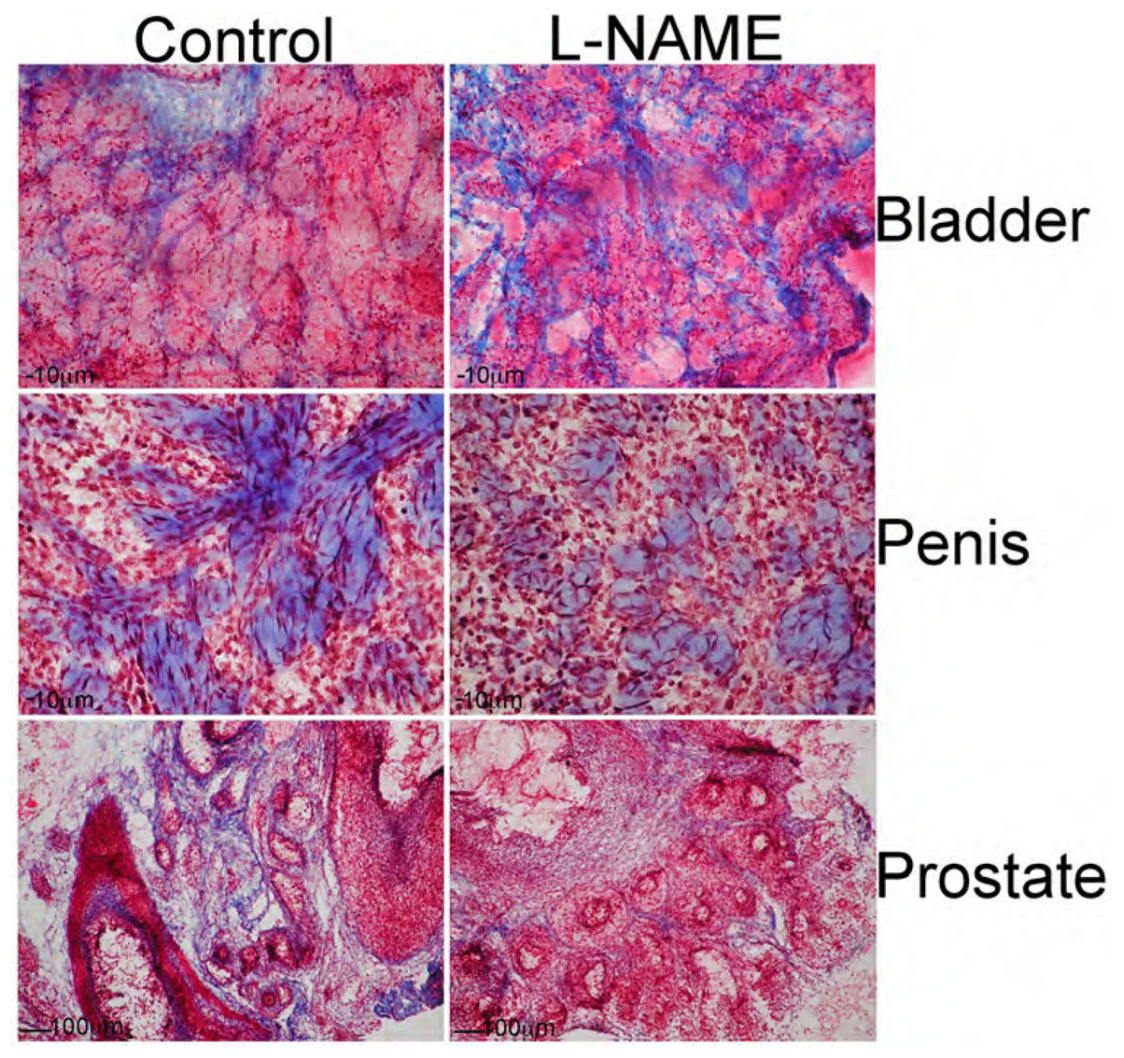
P4 Sprague Dawley rats were treated with the NOS inhibitor L-NAME, or PBS (control), for eight days and bladder, penis, and prostate morphology were examined. In control bladder, smooth muscle bundles interspersed with collagen were observed. With L-NAME treatment the smooth muscle bundles appeared less ordered with increased collagen staining. In control penis, interconnected collagen bundles were interspersed with smooth muscle. With L-NAME treatment the collagen bundles appeared sparse, less interconnected and developed. In the control prostate there were a large number of ducts surrounded by stroma containing collagen. In the L-NAME treated prostate there was less stromal tissue surrounding the ducts, and the ducts appeared larger with increased epithelium. 63–100× magnification.

We examined penis, prostate and bladder morphology in L-NAME treated and control urogenital tissues. The most pronounced change in morphology occurred in the bladder with NOS inhibition. In the control, smooth muscle bundles interspersed with collagen were observed. With L-NAME treatment the smooth muscle bundles appeared less ordered with increased fibrous tissue (Figure 3). In control penis, interconnected collagen bundles were interspersed with smooth muscle (Figure 3). With L-NAME treatment the collagen bundles appeared sparse, less interconnected and developed. The phenotype in the prostate was also well defined. In the control there were a large number of ducts surrounded by stroma containing collagen (blue, Figure 3). However in the L-NAME treated prostate there appeared to be less stromal tissue surrounding the ducts. The ducts appear larger with increased differentiating epithelium.

**Figure 4:**
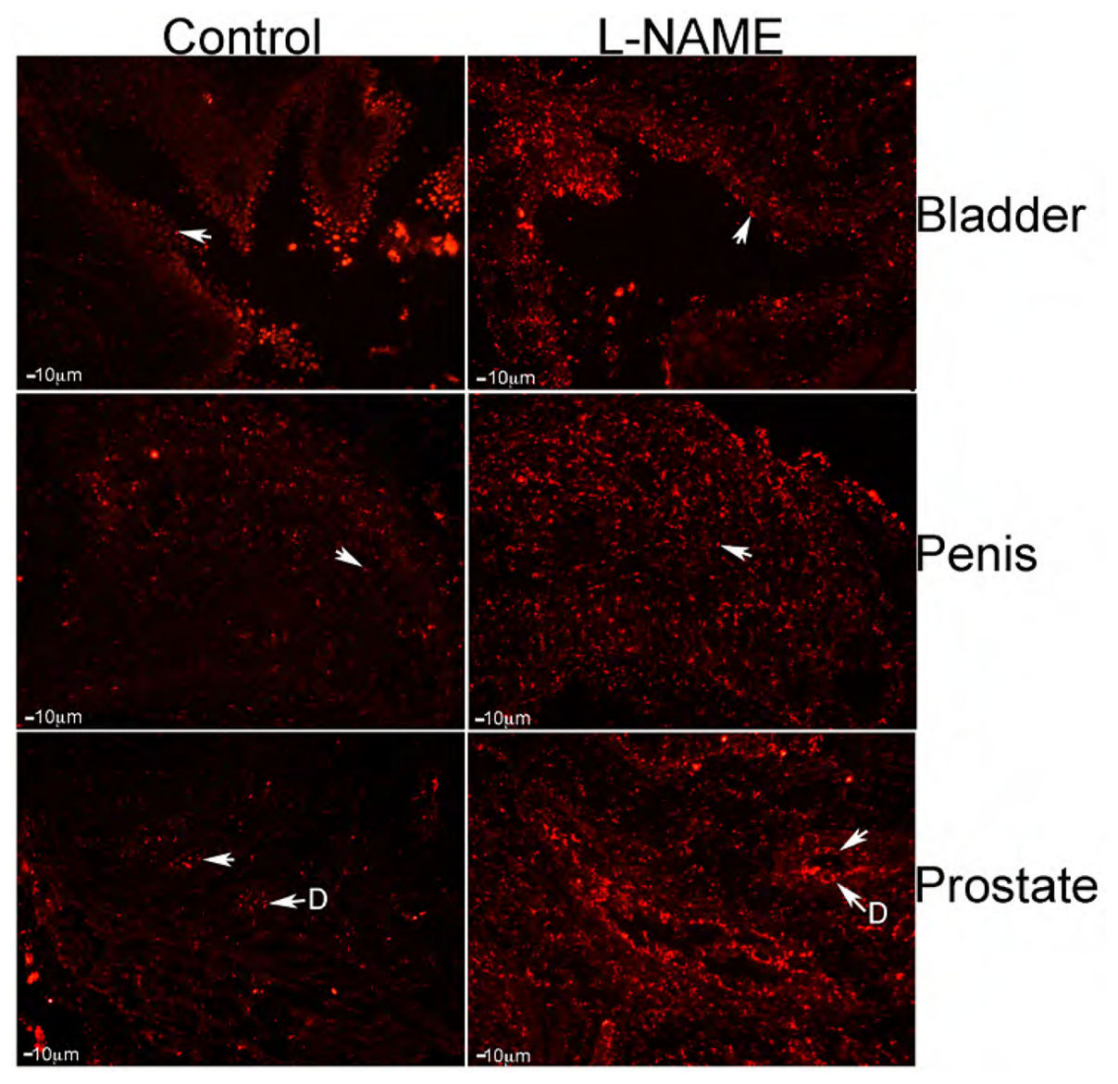
TUNEL assay for apoptosis in control and L-NAME treated bladder, penis, and prostate. Apoptosis appeared elevated by visual observation of the L-NAME treated tissues. Arrows indicate apoptotic cells. 100× magnification. D=Ducts.

Analysis of apoptosis was performed in L-NAME and control penis, prostate and bladder. Apoptosis was occurring at a low level in the control bladder however by visual observation; apoptosis appeared increased in the L-NAME treated tissue (Figure 4). By visual observation, it appeared that apoptosis was more abundant in the L-NAME treated penis in comparison to controls (Figure 4). Apoptosis was identified in the ducts of the control prostate, however abundance of apoptosis appeared increased in response to L-NAME treatment (Figure 4). Apoptosis was increased in all three organs by visual observation, in response to L-NAME treatment.

**Figure 5:**
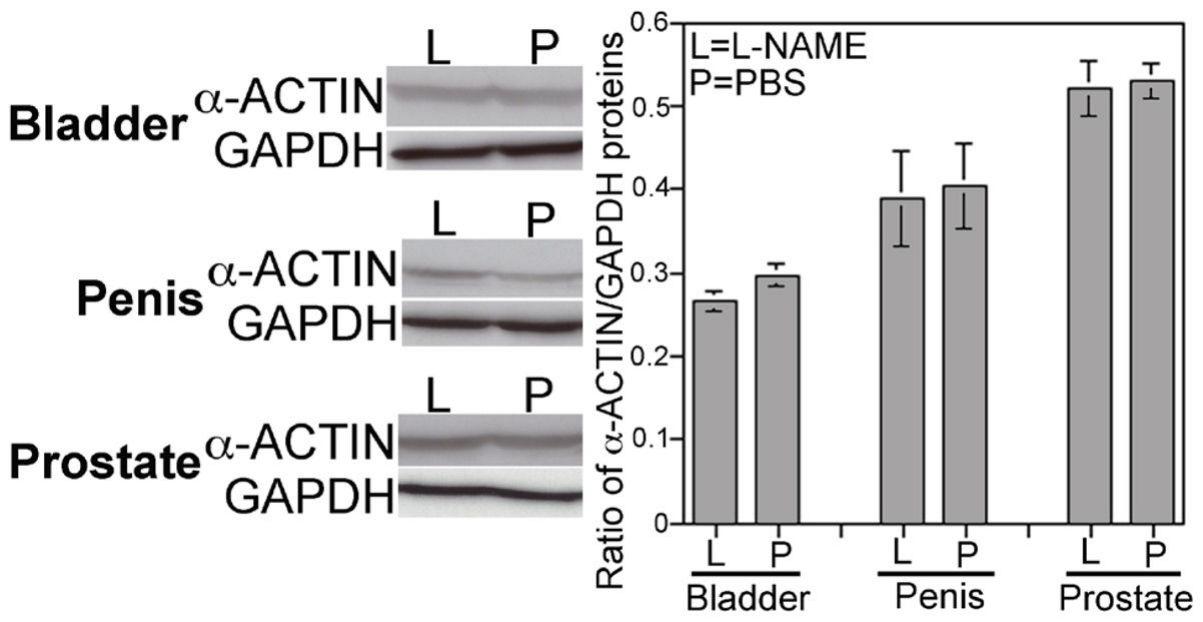
Western analysis of α-ACTIN/GAPDH in control and L-NAME treated bladder, penis and prostate. While a trend towards increased smooth muscle was observed in L-NAME treated bladder, it did not reach statistical significance (p=0.067). Smooth muscle remained unchanged with L-NAME treatment in the developing penis and prostate p=0.418 and 0.435, respectively).

α-ACTIN/GAPDH was quantified by Western analysis in control (n=4) and L-NAME treated (n=4) bladder, penis and prostate in order to quantify changes in smooth muscle. α-ACTIN abundance was not changed with L-NAME treatment in the penis and prostate (p=0.418 and 0.435, respectively) (Figure 5). A trend towards increased α-ACTIN was identified in L-NAME treated bladder however it did not reach statistical significance (p=0.067, Figure 5).

**Figure 6:**
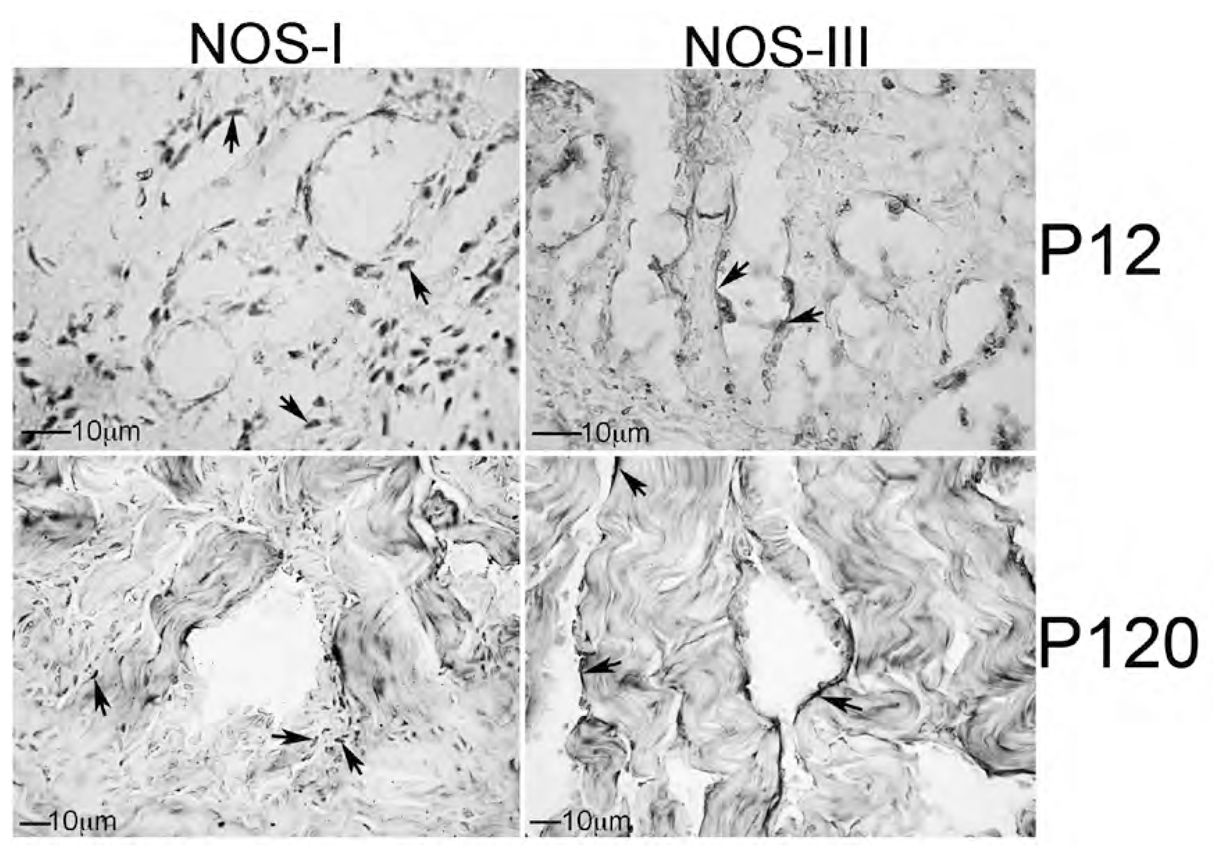
Immunohistochemical analysis of NOS-I and III proteins in P12 and P120 penis. NOS-I protein was localized in nerves interspersed throughout the corpora cavernosa and nerve endings present under the sinusoidal smooth muscle. NOS-III protein was restricted to the endothelium of the developing sinuses. 250–400× magnification. Arrows indicate NOS-I and III staining.

**Figure 7:**
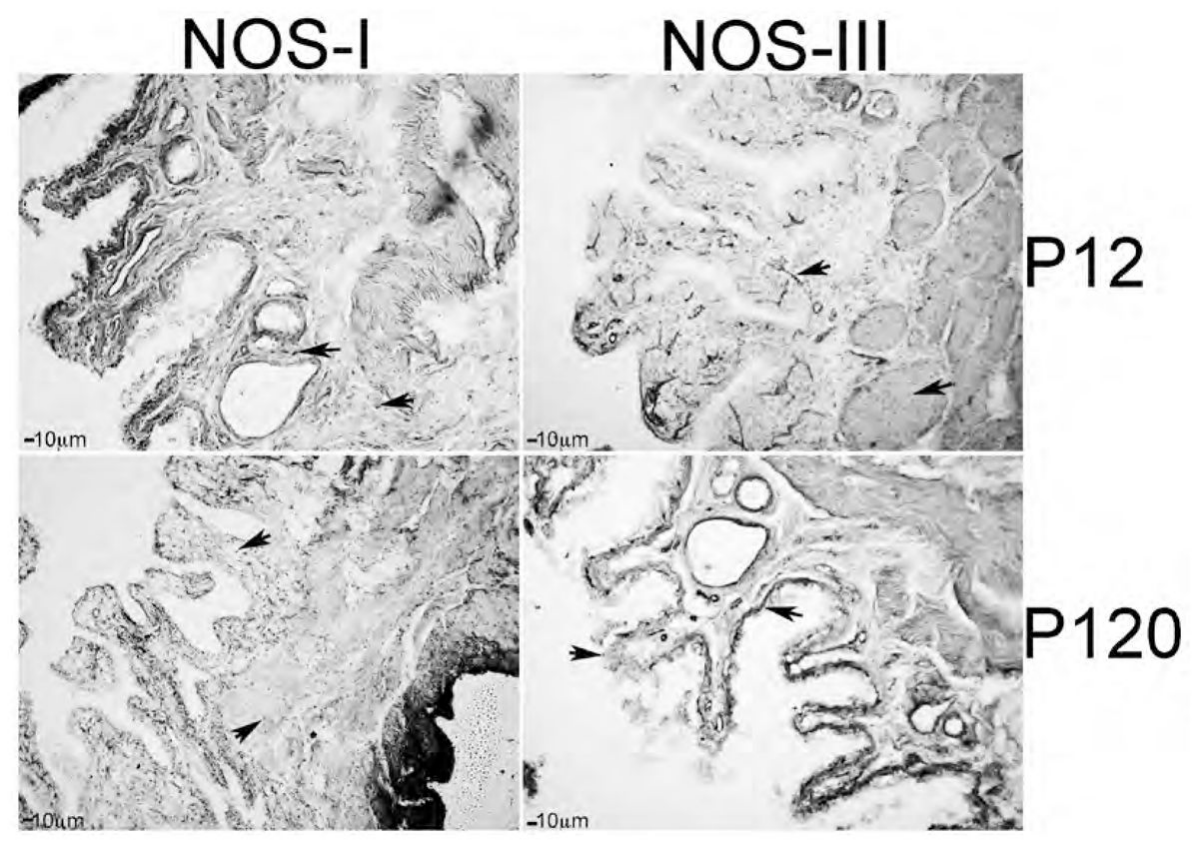
Immunohistochemical analysis of NOS-I and III proteins in P12 and P120 bladder. At P12 NOS-I was abundant in nerves in the urothelium but was also identified in the smooth muscle. In the adult NOS-I was abundant in the smooth muscle as well as the urothelium. NOS-III was localized primarily in the developing basement membrane but was also identifiable in blood vessels at P12 and P120. 100× magnification. Arrows indicate NOS-I and III staining.

**Figure 8:**
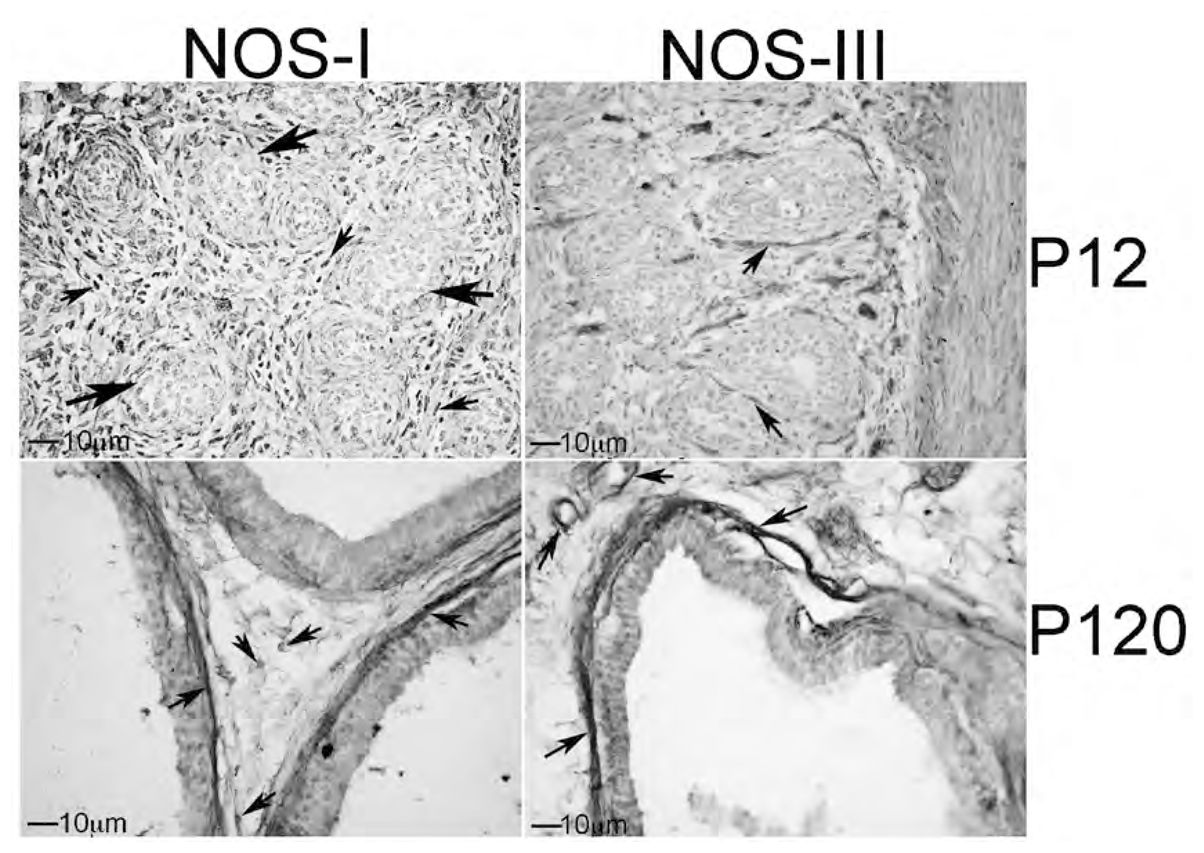
Immunohistochemical analysis of NOS-I and NOS-III proteins in P12 and P120 prostate. At P12, NOS-I was identified in the mesenchyme in between the prostatic ducts while NOS-III was present in the basement membrane surrounding the ductal epithelium. In the adult prostate, NOS-I was abundant in neurons in the stroma between the ducts and in the basement membrane. At P12 and P120, NOS-III was restricted to the basement membrane surrounding the ductal epithelium and in the lining of blood vessels. 250× magnification. Small arrows indicate NOS-I and III staining. Large arrows indicate developing ducts of the prostate.

IHC analysis of NOS-I and III in developing penis, prostate and bladder: We examined the localization of NOS-I and III by IHC analysis of P12 (n=4) and adult P120 (n=4) Sprague Dawley rat penis, prostate and bladder. In the penis, NOS-I was identified in neural tissue innervating the corpora cavernosa (nerve endings) while NOS-III was localized in sinusoidal endothelium (Figure 6). In the adult, NOS-I is more difficult to visualize however it remained localized in nerve endings present under the sinusoidal smooth muscle and in the corpora cavernosal tissue between sinuses (Figure 6). NOS-III remained abundant in the sinusoidal endothelium (Figure 6). In the developing bladder, NOS-I was abundant in the urothelium. NOS-I was also identifiable but less abundant in the smooth muscle. NOS-III was localized primarily in the developing basal urothelial cells (Figure 7). In the adult bladder, NOS-I remained abundant in the urothelium however the smooth muscle appeared more densely innervated since it stained more highly for NOS-I (Figure 7). NOS-III protein was restricted to basal urothelial cells and blood vessels interspersed throughout the urothelium (Figure 7). In the developing prostate, NOS-I was identified in the mesenchyme in between the prostatic ducts while NOS-III was present in the basement membrane surrounding the ductal epithelium and in the lining of blood vessels (Figure 8). In the adult prostate, NOS-I was abundant in neurons in the stroma between the ducts and in the basement membrane, while NOS-III was restricted to the basement membrane surrounding the ductal epithelium and in the lining of blood vessels (Figure 8).

### Time course of Nos III expression in developing penis and prostate

**Figure 9:**
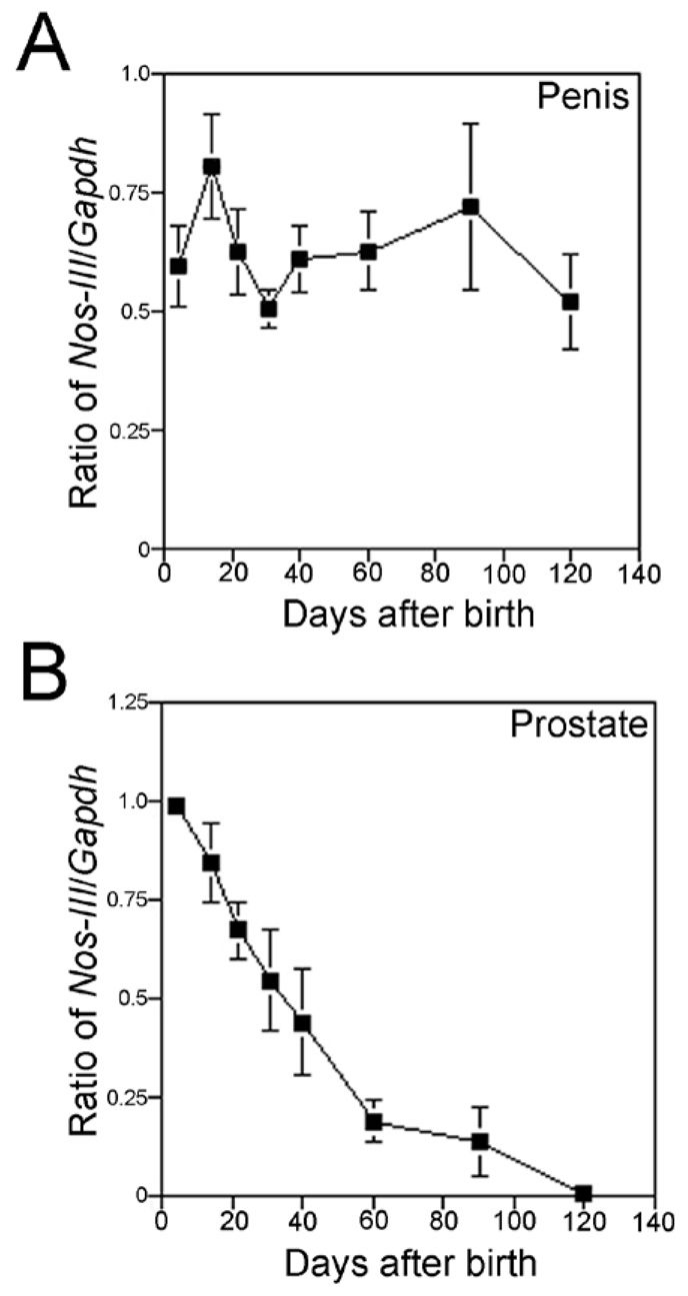
Quantification of Nos-III/Gapdh expression by semi-quantitative RT-PCR in the developing prostate and penis. (A) Nos-III expression was most abundant between one and two weeks after birth when sinusoidal differentiation occurred in the penis, however expression remained high in the adult. (B) Nos-III expression was most abundant in the prostate immediately after birth and expression slowly declined with age.

Nos-III is the most abundant Nos isoform present in the adult prostate and penis so we quantified Nos-III expression in the penis and prostate during development [5,15]. In the penis, Nos-III expression spiked within the first two weeks after birth when postnatal differentiation of the penis was taking place (Figure 9). However, Nos-III remained abundant into the adult period, where it functions as an important regulator of smooth muscle relaxation necessary for erection. In the prostate, Nos-III was most abundant immediately after birth and steadily decreased until adulthood (Figure 9).

### Quantification of NOS-I in the PG/CN after crush injury

**Figure 10:**
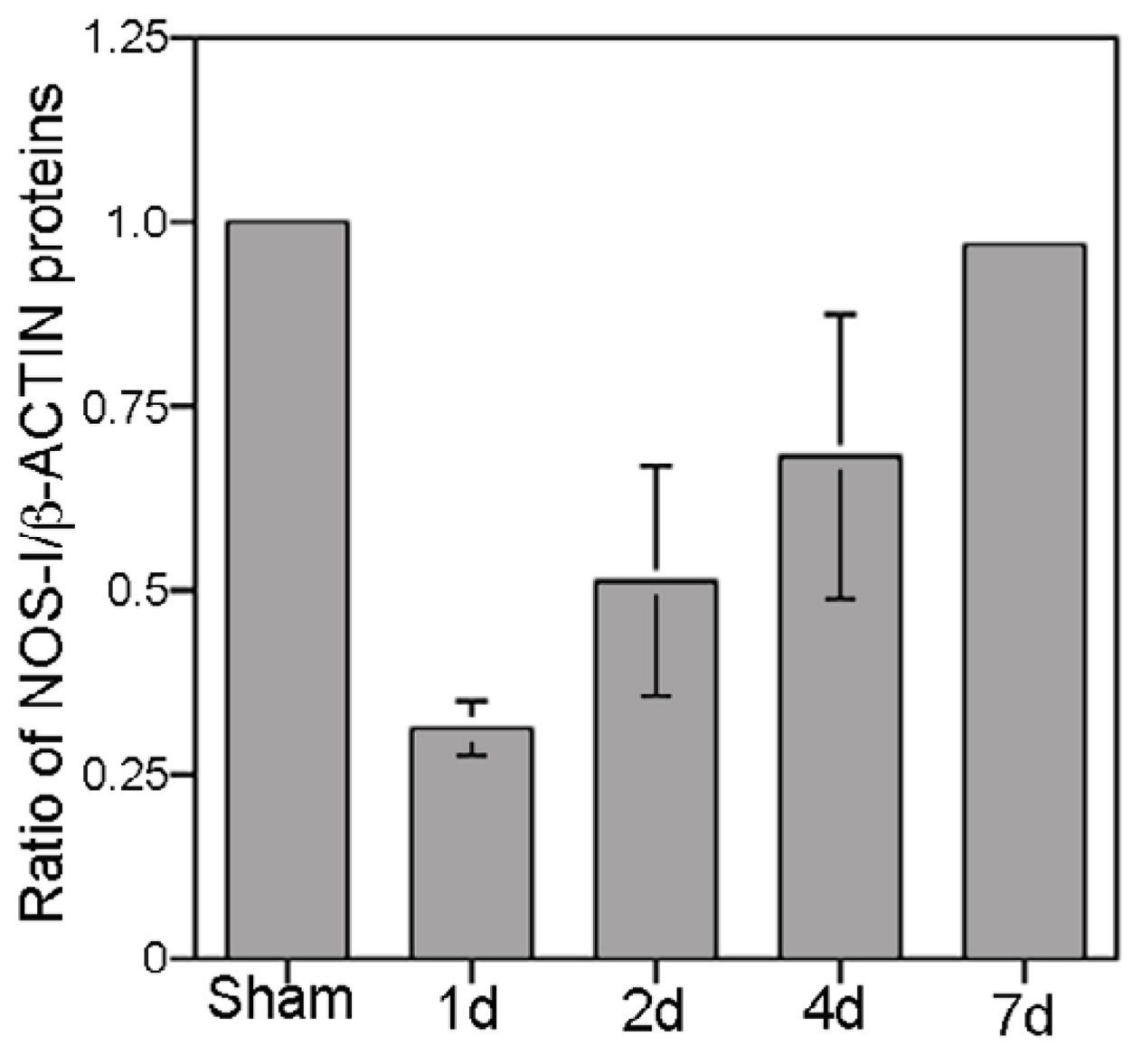
Quantification of NOS-I/β-ACTIN protein by Western analysis in sham and CN crushed PG/CN in the first week after crush injury. NOS-I decreased in the first day after injury and recovered back to normal levels by 7 days after injury. D=day.

We examined the abundance of NOS-I protein in the PG/CN with crush injury. Western analysis showed decreased NOS-I/β-ACTIN in the PG/CN the first day after injury, however, NOS-I/β-ACTIN recovered back to normal levels by one week after bilateral crush injury (Figure 10).

## Discussion

In normal tissues, NOS-I regulates NOS-III synthesis of NO in the endothelium. Since NOS-I is decreased in response to nerve injury, then NOS-III-dependent NO synthesis is correspondingly decreased in the endothelium. Interaction between the endothelium and smooth muscle is critical to maintain penile architecture, and interruption of normal signaling paradigms between the tissues results in apoptosis and remodeling of the corpora cavernosa [30]. Very little is known about how smooth muscle-endothelial interactions are regulated and maintained in the penis and other urogenital organs. When the CN is injured, NOS-I is decreased in the penis and the CN, and this accompanies significant apoptosis in the first week after CN injury suggesting a role for NO signaling between the tissue layers [34].

In this study, we’ve shown during development that NOS plays a previously unsuspected role in organization of the developing urogenital tissues. This novel finding is supported by previous studies, which show that NOS-I is responsive to SHH signaling in the adult penis [33]. Since SHH is a critical mediator of urogenital organ development including the prostate and penis, and a significant regulator of penile smooth muscle, SHH regulation of NOS supports a role for NOS in tissue sculpting and smooth muscle-endothelial interactions [1,2]. A large amount of growth and differentiation occurs in the postnatal period after birth for urogenital organs, and these studies emphasize the importance of nerve integrity for development and maintenance of tissue morphology.

We examined the effect of a non-specific NOS inhibitor, L-NAME, on urogenital organ morphology during postnatal development in order to determine if NOS is a regulator of tissue architecture in addition to its function in smooth muscle relaxation. The result of this type of experiments is more easily interpretable during development rather than in the adult since NOS is more highly expressed during development in the urogenital tissues. Also the additional complication of smooth muscle relaxation effects on tissue oxygenation and composition do not occur prior to erectile capacitance. The morphology of all three urogenital organs was altered with L-NAME treatment. In the bladder, smooth muscle and collagen appear less ordered with more intense/increased collagen staining. NOS-I was localized in the urothelium during development but was more abundant in smooth muscle in the adult. This may reflect NOS’s role in micturition in the adult and may explain why sildenafil is effective in inducing in vitro bladder neck smooth muscle relaxation [35]. In humans, NOS containing nerves form a very minor portion of the total innervation to the bladder at 13 weeks gestation, however with increasing gestational age; NOS-containing nerves are more numerous in the lower urinary tract, the majority occurring at the bladder neck and around the prostatic urethra [36]. The possible role of NOS alterations effecting LUTS development is supported by the known increased autonomic hyperactivity occurring in systemic disorders which affect the bladder and prostate (e.g. diabetes, obesity), and the observed improvement in LUTS with phosphodiesterase type 5 inhibitor use without a change in flow rate [14,15,37–39].

The morphology of the prostate was also altered with L-NAME treatment. There was more collagen (blue) staining observed between the ducts, although the overall prostate weight did not change. NOS-I localization was very different in the developing and adult prostate, which is interesting since localization often offers insight about function. NOS-I protein was localized primarily between the developing ducts at P12 however in the adult NOS-I was abundant in neurons and nerve bundles and the basement membrane adjacent to the columnar epithelium of the ducts. NOS-III was localized in the basement membrane of both the developing and adult prostate. Basement membrane is a layer of specialized extracellular matrix that surrounds normal prostate glands and preserves tissue integrity. Basement membrane is important for keeping tumor cells from spreading and discontinuity of the basement membrane is a prerequisite for tumor cell invasion into interstitial spaces, thus favoring metastasis [39]. Therefore, basement membrane maintenance represents a barrier against cancer development and progression [39]. Since NOS-I and III are abundant in basement membrane, and basement membrane integrity is critical for prostate cancer progression, a potential role for NOS in maintaining prostate morphology is relevant.

Morphology changes in the penis with L-NAME treatment were more subtle than those observed in the bladder and prostate. In the penis, collagen appeared as discrete bundles that were less interconnected and were less developed into a functional network. Whether collagen sub-type remodeling is occurring in response to L-NAME treatment, in addition to gross architectural changes, is interesting however is beyond the scope of this study for analysis. NOS-I protein appeared more abundant in nerve endings within the corpora cavernosa during development, supporting a role for NOS and innervations in development and growth of the penis. Since L-NAME treatment affected collagen morphology, it may have direct implication for penile morphology in response to CN injury where NOS-I is decreased in both the CN and penis. Decreased NOS-I, which occurs frequently with prostatectomy and in diabetic animal models, leads to ED [5,8]. Suggesting that in addition to the known neural relaxation role of NOS-I, our findings support a role for NOS-I having a morphologic regulatory role.

These studies revealed a 25% increase in bladder weight with L-NAME treatment. Does this change result from increased smooth muscle, collagen or both? We observed a trend (11%) towards increased smooth muscle in L-NAME treated bladder that did not reach significance (p=0.067). It is likely that a higher number of rats would be required to quantify such a small change in morphology. It is also possible that the cell type that increased was not fully differentiated, so was not quantifiable by α-ACTIN analysis. In the literature it has been shown that long term L-NAME treatment of the adult penis leads to fibrosis and that L-NIL treatment (selective inhibitor of NOS-II) increased collagen deposition in a manner similar to the Peyronie’s fibrotic plaque, thus suggesting a link between altered NOS and collagen regulation [11]. The observed increase in bladder weight with L-NAME treatment in the absence of increased smooth muscle supports a role for NOS in collagen regulation. It is also possible that L-NAME exposure increased fibroblasts and thus increased bladder weight. This is commonly observed in human bladders damaged via obstruction, and in certain neurogenic bladders, which show a thickened bladder wall, increased matrix, fibroblasts/fibrocytes and loss of functional muscle. This idea is supported by inhibited NOS signaling in cardiomyocytes, which leads to fibrosis [40]. It is important to note that high dose L-NAME treatment can disturb growth by impacting testicular function, and thus affecting the testosterone axis [41]. However at the 50 mg/kg/day concentration used in this study, previous investigators have demonstrated that there is no change in testosterone abundance [41].

NOS-I protein decreases in the PG/CN when the CN is crushed, simulating the injury that occurs during prostatectomy, and recovery of NOS-I in the PG/CN occurs within a week after crush injury. Decreased NOS-I in the penis occurs as a result of decreased NOS-I in the PG/CN, when the CN is crushed [5]. Increased apoptosis, primarily in penile smooth muscle, occurs as early as 1 day after CN injury and decreases substantially by a week after injury, with peak between 2 and 4 days [34,42]. While NOS-I recovers in the CN, the downstream morphological changes in the penis are not so easily reversed and significantly affect erectile function. Based on our findings of NOS contribution to urogenital morphogenesis, it is possible that decreased NOS-I contributes to the CN injury induced morphological changes in the penis, which may explain why long term treatment of prostatectomy patients with phosphodiesterase type 5 inhibitors (increase available NOS) improves post prostatectomy erectile function.

## Conclusions

These results show that NOS is important for normal urogenital development. Since NOS is decreased in ED models, it may contribute to the abnormal morphology observed in ED patients and animal models.

## Figures and Tables

**Table 1:** Average penis, prostate and bladder weights ± standard error of the mean.

	Control (n=4)	L-NAME (n=4)
**Penis**	0.036 ± 0.002	0.0385 ± 0.001
**Prostate**	0.002 ± 0.002	0.0238 ± 0.003
**Bladder**	0.040 ± 0.003	0.0535 ± 0.003*

* p=0.018

## References

[1] Podlasek CA, Barnett DH, Clemens JQ, Bak PM, Bushman W (1999). Prostate development requires Sonic hedgehog expressed by the urogenital sinus epithelium. Dev Biol.

[2] Podlasek CA, Zelner DJ, Jiang HB, Tang Y, Houston J (2003). Sonic hedgehog cascade is required for penile postnatal morphogenesis, differentiation, and adult homeostasis. Biol Reprod.

[3] Burnett AL, Lowenstein CJ, Bredt DS, Chang TS, Snyder SH (1992). Nitric oxide: a physiologic mediator of penile erection. Science.

[4] Burnett AL, Tillman SL, Chang TS, Epstein JI, Lowenstein CJ (1993). Immunohistochemical localization of nitric oxide synthase in the autonomic innervation of the human penis. J Urol.

[5] Podlasek CA, Gonzalez CM, Zelner DJ, Jiang HB, McKenna KE (2001). Analysis of NOS isoform changes in a post radical prostatectomy model of erectile dysfunction. Int J Impot Res.

[6] El-Sakka AI, Lin CS, Chui RM, Dahiya R, Lue TF (1999). Effects of diabetes on nitric oxide synthase and growth factor genes and protein expression in an animal model. Int J Impot Res.

[7] Akingba AG, Burnett AL (2001). Endothelial nitric oxide synthase protein expression, localization, and activity in the penis of the alloxan-induced diabetic rat. Mol Urol.

[8] Podlasek CA, Zelner DJ, Bervig TR, Gonzalez CM, McKenna KE (2001). Characterization and localization of nitric oxide synthase isoforms in the BB/WOR diabetic rat. J Urol.

[9] Bivalacqua TJ, Champion HC, Mehta YS, Abdel-Mageed AB, Sikka SC (2000). Adenoviral gene transfer of endothelial nitric oxide synthase (eNOS) to the penis improves age-related erectile dysfunction in the rat. Int J Impot Res.

[10] Vernet D, Ferrini MG, Valente EG, Magee TR, Bou-Gharios G (2002). Effect of nitric oxide on the differentiation of fibroblasts into myofibroblasts in the Peyronie’s fibrotic plaque and in its rat model. Nitric Oxide.

[11] Ferrini MG, Vernet D, Magee TR, Shahed A, Qian A (2002). Antifibrotic role of inducible nitric oxide synthase. Nitric Oxide.

[12] Sezen SF, Lagoda G, Burnett AL (2012). Neuronal nitric oxide signaling regulates erection recovery after cavernous nerve injury. J Urol.

[13] Bloch W, Klotz T, Loch C, Schmidt G, Engelmann U (1997). Distribution of nitric oxide synthase implies a regulation of circulation, smooth muscle tone, and secretory function in the human prostate by nitric oxide. Prostate.

[14] McVary K (2006). Lower urinary tract symptoms and sexual dysfunction: epidemiology and pathophysiology. BJU Int.

[15] Podlasek CA, Bond CW, Tang Y, Marr L, Angeloni N (2012). Analysis of changes in nitric oxide synthase signaling in BB/WOR diabetic prostate. Andrology-Open Access.

[16] Gradini R, Realacci M, Ginepri A, Naso G, Santangelo C (1999). Nitric oxide synthases in normal and benign hyperplastic human prostate: immunohistochemistry and molecular biology. J Pathol.

[17] Chertin B, Rolle U, Solari V, Cascio S, Puri P (2004). The role of nitric oxide in bladder urothelial injury after bladder outlet obstruction. BJU Int.

[18] Saito M, Miyagawa I (2002). N(G)-nitro-L-arginine methylester, a nitric oxide synthase inhibitor, diminishes apoptosis induced by ischemia-reperfusion in the rat bladder. Neurourol Urodyn.

[19] Quintana-Lopez L, Blandino-Rosano M, Perez-Arana G, Cebada-Aleu A, Lechuga-Sancho A (2013). Nitric oxide is a mediator of antiproliferative effects induced by proinflammatory cytokines on pancreatic beta cells. Mediators Inflamm.

[20] Callsen-Cencic P, Mense S (1997). Expression of neuropeptides and nitric oxide synthase in neurones innervating the inflamed rat urinary bladder. J Auton Nerv Syst.

[21] Ozawa H, Chancellor MB, Jung SY, Yokoyama T, Fraser MO (1999). Effect of intravesical nitric oxide therapy on cyclophosphamide-induced cystitis. J Urol.

[22] Persson K, Igawa Y, Mattiasson A, Andersson KE (1992). Effects of inhibition of the L-arginine/nitric oxide pathway in the rat lower urinary tract in vivo and in vitro. Br J Pharmacol.

[23] Gillespie JI, Markerink-van Ittersum M, de Vente J (2005). Expression of neuronal nitric oxide synthase (nNOS) and nitric-oxide-induced changes in cGMP in the urothelial layer of the guinea pig bladder. Cell Tissue Res.

[24] De Ridder D, Roskams T, Van Poppel H, Baert L (1999). Nitric oxide synthase expression in neurogenic bladder disease: a pilot study. Acta Neurol Belg.

[25] Ozkara H, Alan C, Atukeren P, Uyaner I, Demirci C (2006). Changes of nitric oxide synthase-containing nerve fibers and parameters for oxidative stress after unilateral cavernous nerve resection or manuplation in rat penis. Chin J Physiol.

[26] Mullerad M, Donohue JF, Li PS, Scardino PT, Mulhall JP (2006). Functional sequelae of cavernous nerve injury in the rat: is there model dependency. J Sex Med.

[27] Nangle MR, Keast JR (2007). Reduced efficacy of nitrergic neurotransmission exacerbates erectile dysfunction after penile nerve injury despite axonal regeneration. Exp Neurol.

[28] Angeloni NL, Bond CW, Tang Y, Harrington DA, Zhang S (2011). Regeneration of the cavernous nerve by Sonic hedgehog using aligned peptide amphiphile nanofibers. Biomaterials.

[29] Takeda M, Tang R, Shapiro E, Burnett AL, Lepor H (1995). Effects of nitric oxide on human and canine prostates. Urology.

[30] Podlasek CA, Meroz CL, Tang Y, McKenna KE, McVary KT (2007). Regulation of cavernous nerve injury-induced apoptosis by sonic hedgehog. Biol Reprod.

[31] Angeloni NL, Bond CW, Monsivais D, Tang Y, Podlasek CA (2009). The role of hedgehog-interacting protein in maintaining cavernous nerve integrity and adult penile morphology. J Sex Med.

[32] Leeson TS, Leeson CR (1966). Penile cavernous tissue: an electron microscopic study of its development in the rat. Acta Anat (Basel).

[33] Podlasek CA, Meroz CL, Korolis H, Tang Y, McKenna KE (2005). Sonic hedgehog, the penis and erectile dysfunction: a review of sonic hedgehog signaling in the penis. Curr Pharm Des.

[34] User HM, Hairston JH, Zelner DJ, McKenna KE, McVary KT (2003). Penile weight and cell subtype specific changes in a post-radical prostatectomy model of erectile dysfunction. J Urol.

[35] Bittencourt JA, Tano T, Gajar SA, Resende AC, de Lemos Neto M (2009). Relaxant effects of sildenafil on the human isolated bladder neck. Urology.

[36] Dixon JS, Jen PY (1995). Development of nerves containing nitric oxide synthase in the human male urogenital organs. Br J Urol.

[37] Mouli S, McVary KT (2009). PDE5 inhibitors for LUTS. Prostate Cancer Prostatic Dis.

[38] Gacci M, Corona G, Salvi M, Vignozzi L, McVary KT (2012). A systematic review and meta-analysis on the use of phosphodiesterase 5 inhibitors alone or in combination with α-blockers for lower urinary tract symptoms due to benign prostatic hyperplasia. Eur Urol.

[39] Gandellini P, Profumo V, Casamichele A, Fenderico N, Borrelli S (2012). miR-205 regulates basement membrane deposition in human prostate: implications for cancer development. Cell Death Differ.

[40] Kazakov A, Hall R, Jagoda P, Bachelier K, Müller-Best P (2013). Inhibition of endothelial nitric oxide synthase induces and enhances myocardial fibrosis. Cardiovasc Res.

[41] Haluzík M, Nedvídková J, Kopský V, Jahodová J, Horejsí B (1998). The changes of the thyroid function and serum testosterone levels after long-term L-NAME treatment in male rats. J Endocrinol Invest.

[42] Angeloni N, Bond CW, Harrington D, Stupp S, Podlasek CA (2013). Sonic hedgehog is neuroprotective in the cavernous nerve with crush injury. J Sex Med.

